# Features of the UK childcare environment and associations with preschooler's in-care physical activity

**DOI:** 10.1016/j.pmedr.2015.12.004

**Published:** 2015-12-17

**Authors:** Kathryn R. Hesketh, Esther M.F. van Sluijs

**Affiliations:** MRC Epidemiology Unit & Centre for Diet and Activity Research (CEDAR), University of Cambridge School of Clinical Medicine, Box 285 Institute of Metabolic Science, Cambridge Biomedical Campus, Cambridge CB2 0QQ, United Kingdom

**Keywords:** PA, Physical activity, SED, Sedentary, LPA, Light physical activity, MVPA, Moderate-to-vigorous physical activity, ICC, Intra-class correlation co-efficient, SPACE, Studying Physical Activity in preschool-aged Children and their Environment Study, IMD, Index of Multiple Deprivation, EPAO, Environment Policy Assessment and Observation, Ofsted, Office for Standards in Education, Preschool-aged children, Physical activity, Childcare, Sedentary, Policy, Environment

## Abstract

**Objective:**

Features of the childcare environment may influence children's in-care physical activity (PA). We assessed the association between UK preschool care-provider, environmental and policy factors and 3–4-year-olds' average daily in-care sedentary behaviour (SED) and PA.

**Methods:**

In 2013, we used accelerometers to measure the in-care SED/ PA of 201 3–4-year-old children (51% female) in 30 preschools in Cambridgeshire, UK, (average wear time: (mean ± SD) 4.2 ± 1.3 week-days). We assessed the childcare environment using the Environment and Policy Assessment and Observation tool; demographic and carer information was taken from questionnaires. We used three-level mixed-effects regression analyses (adjusted for sex, in-care time and travel mode to care) to determine the association between childcare factors and children's in-care average daily minutes/hour spent SED, in light PA (LPA) and in moderate-to-vigorous PA (MVPA).

**Results:**

Children spent 5.6 ± 2.5 h in care per day on average; clustering of PA within preschools was limited (ICCs: 0.003–0.05). Fully adjusted models showed that active opportunities were positively associated with children's in-care SED. No associations with in-care LPA and MVPA were observed.

**Conclusion:**

Few care-provider, environmental and policy factors were associated with children's in-care activity. UK childcare policies advocating child-driven play, moving freely indoors and outdoors, may be more conducive to individual children's PA.

## Highlights

• Little is known about what influences children's activity in UK childcare settings.
• In 30 settings, 201 3–4-year-olds provided valid in-care objective activity data.
• We assessed 23 potential care-provider, environmental and policy correlates.
• Few associations were observed with children's in-care sedentary/physical activity.
• UK preschool policies may be more conducive to individual activity preferences.

## Background

As the time children spend in out-of-home care increases, the childcare environment is likely to exert a greater influence on young children's activity (Ward et al., 2010). Guidelines for under-5 s recommend 180 min of total activity daily (Department of Health, 2011, Tremblay et al., 2012), including light (LPA; e.g. crawling, walking) and moderate-to-vigorous physical activity (MVPA; e.g. running, jumping). Yet low levels of MVPA (Tucker, 2008) in combination with high levels of sedentary behaviour appear common during the childcare day (Reilly, 2010).

Much of the evidence regarding levels of preschool-aged children's activity in childcare comes from the USA and mainland Europe (Trost et al., 2010) (where ‘preschool’ is defined as 2.5/3–5/6 years depending on country (The World Bank, 2013)). Positive associations with preschool-aged children's physical activity have been reported for fixed (e.g. climbing frames) and portable (e.g. wheeled) toys, the presence of natural elements (e.g. vegetation), and staff education, training and behaviour in the playground (Trost et al., 2010). In contrast, qualitative work suggests that factors including parental concerns about child safety and emphasis on educational outcomes (Copeland et al., 2012) may result in greater sedentary behaviour. The childcare day in the United States (US), and to a lesser extent in mainland Europe (Raustrop et al., 2012, Cardon and De Bourdeaudhuij, 2008), tends to include structured periods of learning and recess. In the United Kingdom (UK), settings operate a free-flow policy where regardless of weather conditions children self-select activities, both inside and out, for the majority of the day. Understanding how these contextual differences and elements in the UK childcare environment influence preschoolers' physical activity may be beneficial to inform research and practitioners internationally.

This study therefore sought to determine whether elements in the interpersonal, environmental and policy domains are associated with UK 3–4-year-old children's sedentary behaviour and physical activity when in childcare.

## Methods

### Study design and recruitment

Data were from the “Studying Physical Activity in preschool-aged Children and their Environment (SPACE) Study” (Hesketh et al., 2015). Both preschool (state-run education) and nursery (privately-run care) ‘settings’ were purposively recruited to enable comparison, as they are (usually) differentially funded, operate in different built environments and vary in the care provided (see Table 1). Recruitment and data collection took place in January–July 2013. Detailed information about setting and child recruitment has been published elsewhere (Hesketh et al., 2015). Briefly, 88 settings in Cambridgeshire were approached to participate; 30 (34%) setting managers provided written consent. Within settings, preschool-aged children were eligible to participate (n = 602) if they: were 3–4-years-old; would be present on the designated measurement day; were free from physical disability; and attended the setting for at least 9 h per week. Parents/guardians provided written consent; children provided verbal assent prior to measurement. A minimum of 5 participating children per setting was required to ensure sufficient analytical power. The University of Cambridge Psychology Ethics Committee provided ethical approval for the study (Pre.2012.68).

### Data collection

At settings, we fitted children with an Actiheart activity monitor (Cambridge Neurotechnology Ltd, UK), a combined lightweight heart-rate monitor and accelerometer, previously validated in preschool-aged children (Adolph et al., 2012). The unit was secured to the chest, and set to record at 15-second epochs. Written instructions were sent home to the parents, together with a previously validated questionnaire (McMinn et al., 2009) designed to assess potential correlates of physical activity. We encouraged children to wear the monitor continuously for < 7 days, including during water-based activity and sleep.

### Outcome variables

Counts data from Actiheart monitors were downloaded and processed using STATA 13/SE. Childcare attendance during the measurement week was reported by parents using a specially designed open-ended question (Hesketh et al., 2015). To reflect when children were most likely to be active and/or in care, we restricted data to between 7 am and 6 pm (maximum 660 min). Although children would plausibly be awake outside these hours, they were not, according to parental report, in care. We removed data periods of > 100 min of zero-activity counts (Collings et al., 2013), and days with < 600 min of recording (Beets et al., 2011) (average in-care days: (mean ± SD) 4.2 ± 1.3 days). We applied a previously validated conversion factor (Ridgway et al., 2011), and used validated cut points (Pate et al., 2006) to classify children's activity as sedentary (SED: < 38 Actigraph counts per 15 s); LPA (> 38–420); and MVPA (> 421) (Pate et al., 2006). Each child's activity and location data were matched in 15-minute segments (Hesketh et al., 2015). Only ‘in care’ segments were used in the present analyses; outcome measures were expressed as average daily minutes per hour spent SED, in LPA and MVPA.

#### Exposure measures

A trained researcher assessed the setting environment using the validated Environment Policy Assessment and Observation (EPAO) tool (Ward et al., 2008). Responses to questions across 8 physical activity sub-domains from the EPAO were scored from 0 to 2 and totalled within a given domain to a possible maximum of 20 points, yielding 8 physical activity subscale scores (Bower et al., 2008). An overall physical activity environment score (possible range 0–160, higher score indicates more supportive environment) was also calculated for each setting (‘EPAO score’).

Additional exposure variables were chosen based on prior evidence (Trost et al., 2010). The average time staff had spent at the setting and as a childcare provider was taken from the questionnaire completed by each carer and used to calculate averages for each setting. Setting managers reported daily minutes children spent in gross motor play (in categories: < 60 min; 61–120 min; 121–180 min, > 180 min), and five rules relating to outside play: in light rain, heavy rain, snow, wet conditions and high UV/sun (allowed always; in special clothing; never). Each setting's Office for Standards in Education (Ofsted) rating (satisfactory, good/outstanding), given following independent external review by trained inspectors, was obtained from the Ofsted website (https://www.gov.uk/government/organisations/ofsted).

#### Statistical analyses

All children with > 2 valid week-days of accelerometry data were included in analyses (n = 201), and a pre-defined significance level of *p <* 0.05 was used for all analyses. Descriptive statistics were calculated and compared by setting type using t-tests for normal, Mann–U Whitney for non-normal or χ (Department of Health, 2011) tests for categorical data.

Three-level hierarchical linear regression models were fitted, assessing the associations between childcare-related factors and children's daily average minutes per hour of in-care SED, LPA and MVPA (Level 1: in-care activity; Level 2: child; Level 3: setting). Univarible regression models were first conducted to assess the association between each exposure variable and children's activity. All variables significantly associated in univariable models were subsequently entered into a multivariable regression model. Variables were removed from the adjusted model if they did not meet the pre-defined significance level. All analyses were adjusted for sex, daily hours spent in care and parent-reported travel mode to childcare.

## Results

Thirty settings (15 preschools, 15 nurseries) provided valid observational and questionnaire data (Table 1). Area deprivation scores for participating settings did not differ from those who declined to participate (participating: median 8.3 (Range: 1–27); declined: 8.6 (2–35); Wilcoxon rank-sum test: *p* = 0.48). Compared to care-providers, setting managers were on average older (44.8 (SD: 9.4) vs. 35.4 (12.0) years old) and had worked in childcare for longer (13.5 (8.7) vs. 8.1 (5.8) years).

The mean total EPAO physical activity environment score was 85.9 (SD: 11.6; Range: 58.9–110.2). Mean subscale scores ranged from 4.7 (3.9; 0–20) for physical activity training and education to 15.3 (3.8; 6.7–20) for Active Opportunities; the average subscale score was 10.7 (1.5; 7.4–13.8) across all 8 scales.

Associations between children's in-care activity and the preschool environment

In univariable analyses, four factors were associated with children's in-care SED; no factors were associated with children's in-care LPA and MVPA (Table 2). Only Active Opportunities remained significantly associated with SED in adjusted models. Children's in-care activity did not cluster within setting (intraclass correlation coefficients (ICCs): SED: 0.04; LPA: 0.003; MVPA: 0.05).

## Discussion

This is the first study to investigate associations between factors in the UK childcare environment and preschoolers' physical activity, showing that childcare variables explain little variation in children's activity. Although several interpersonal and environmental-level factors were associated with children's in-care sedentary behaviour in univariable analyses, only one remained in multivariable models. No factors were associated with in-care LPA and MVPA. This suggests the UK childcare environment may have a limited influence on children's activity, being conducive to children's individual activity preferences instead. How individual and unexplored social factors affect children's in-care activity now warrants further investigation, and may be useful when exploring ways to increase activity in lesser active children of this age.

Previous work conducted in the USA, using both direct observation and accelerometers to measure children's activity, showed that children's activity levels were primarily affected by the setting they attended (Pate et al., 2004, Pate et al., 2008). In contrast, children's in-care activity levels appeared not to cluster within settings here, with ICCs of 0.003–0.05 similar to those seen in a Danish study assessing preschoolers' objectively measured in-care activity (Groenholt Olesen et al., 2013). Variation in the childcare day may in part contribute to these differences. Structured periods of play, recess, and group teaching tend to occur in US and mainland European countries (Raustrop et al., 2012). For example, one study comparing differences between children's average activity in US and Swedish childcare centres found US children spent more time indoors, with greater MVPA observed when children were outdoors (Raustrop et al., 2012). In contrast, free-flow policies in the UK encourage children to select their own activities, both inside and out, for the majority of the day. A less structured childcare day may therefore result in the childcare environment exerting a smaller influence on UK children's activity. Given our findings, adoption of a less structured childcare day may therefore be one way for practitioners to positively influence young children's physical activity levels, and may be piloted relatively easily.

Additionally, our research and the Danish study assessed children's individual-level, daily in-care activity and used multi-level analyses to capture the within-child fluctuations, which may better represent children's actual in-care activity levels. We identified larger fluctuations in within-child compared to between-child daily activity (when in care), which may reflect children's self-selection of activities and UK childcare policies. As such, individual and social factors, may therefore be a stronger driver of children's in-care physical activity levels in the UK (Bower et al., 2008, Gubbels et al., 2011).

That few associations were found between childcare-related factors and children's in-care activity here may corroborate this. The EPAO has been used to assess childcare environments in our study, as well as in US (Bower et al., 2008) and Dutch (Gubbels et al., 2011) studies, with similar average subscale scores seen (10.7 vs 10.2 in the US study (Bower et al., 2008); not reported in Dutch study). Only the (unexpected) positive association between increased active opportunities and sedentary time remained significant in adjusted models. In contrast, in a Dutch cohort of 2–3 year olds, EPAO-assessed childcare active opportunities were positively associated with directly-observed higher intensity activity (Gubbels et al., 2011). In the US, children in more supportive childcare environments were shown to have greater active and sedentary opportunities, spend more time in MVPA and less time sedentary (Bower et al., 2008). Notwithstanding the variation in outcome measures used, it is possible that differences in associations seen between these studies are indeed a result of cultural or operational differences in the childcare environment, which the EPAO was not designed to identify.

#### Strengths and limitations

Previous studies assessing the influence of the childcare environment on children's activity have used direct observation or accelerometers to provide an aggregated (childcare-level) overview of children's physical activity levels (Bower et al., 2008, Gubbels et al., 2011). We used an objective measure to capture children's individual-level daily activity, which may reduce potential biases associated with direct observation. Staff were blinded to study aims to minimise bias and avoid behaviour change during the EPAO observation; no staff-related behaviours appeared to influence children's in-care activity here.

Children's actual in-care hours may have varied from those reported, resulting in misclassification of ‘in-care’ time; we adjusted for usual mode of transport to care to account for variation in actual / reported arrival time. Every effort was made to use accelerometry data reported to have occurred at the observed setting, but 7% of children attended two different settings during the measurement week and it was not possible to determine the participating setting. This may have attenuated the association between childcare factors and activity. However, post-hoc sensitivity analyses excluding these children did not alter the overall conclusions. Finally, though heterogeneity in environment scores between settings is similar to those reported previously (Bower et al., 2008), insufficient variation in exposures may have contributed to the limited number of significant associations seen here.

## Conclusion

This is the first work to assess the UK childcare physical activity environment and determine factors associated with children's in-care activity. Children's activity appeared not to cluster by setting, suggesting that the childcare environment may have a limited influence on children's in-care physical activity in the UK. This is supported by the finding that few investigated factors appear to be associated with children's in-care activity behaviour. Other locations or social groupings (e.g. parent-child groups) may prove more appropriate to facilitate and encourage activity amongst UK preschoolers.

## Conflict of interest statement

The authors declare no conflict of interest.

## Figures and Tables

**Table 1:** Characteristics of participating settings by type.

	All settings (n = 30)	Nursery^a^ (n = 15)	Preschool^b^ (n = 15)
*Interpersonal*			
Children enrolled at setting^c⁎^ (mean (SD))	72 (52)	95 (58)	46 (28)
3–4 year-olds enrolled at setting (mean (SD))	44 (30)	49 (33)	38 (25)
Class composition (n (%))			
2–4 year olds	13 (43)	6 (40)	4 (27)
3–4 year olds	17 (57)	9 (60)	11 (73)
% Non-white children (mean (SD))	11.2 (13.6)	15.0 (17.7)	7.4 (6.6)
Government funded places (mean (SD))	33 (24)	27 (15)	37 (30)
Children per staff member^d^ (mean (SD))	3.2 (7.1)	3.2 (9.0)	3.2 (5.6)
Preschool Staff (all mean (SD))			
Age in years	38.9 (8.5)	34.9 (7.9)	43.6 (6.7)
Years at setting	6.3 (3.4)	6.6 (3.7)	6.2 (3.3)
Years in childcare	9.7 (5.3)	8.9 (3.3)	10.8 (6.8)
			
*Environmental*			
Number of hours observed^⁎⁎^ (mean (SD))	7.1 (2.4)	9.1 (1.0)	5.1 (1.5)
Fixed equipment^e^ (mean (SD))	4.8 (1.7)	5.0 (1.7)	4.6 (1.7)
Portable equipment^e^ (mean (SD))	6.1 (1.5)	6.2 (1.6)	6.1 (1.5)
Reported time spent in GMP (n (%))			
0–60 min	4 (13)	2 (13)	2 (13)
61–120 min	8 (27)	2 (13)	6 (40)
121–180 min	7 (23)	3 (20)	4 (27)
> 180 min	11 (37)	8 (53)	3 (20)

GMP: Gross Motor Play; a: Nursery: offers full day care (~ 7 am–6 pm) for children < 1 year up to 4 years 11 months, usually privately run; b: offers sessional care (~ 9 am–12noon and/or 12noon–3 pm) for children between 2 years 9 months and 4 years 11 months old, usually state-run; c: Number of children enrolled at setting includes all children who attend on weekly basis, regardless of age and study eligibility; d: Calculated as a ratio: number of children in room /number of staff in room; e: refers to the average number of pieces of fixed/ portable play equipment visible at setting.

Significant difference by setting type: *p < 0.05; **p < 0.005.

**Table 2:** Associations between children's in-care activity and (elements in) the preschool environment.

Exposure	Outcome [β (95% CI)]					
SED		LPA		MVPA	
Univariable	Multivariable	Univariable	Multivariable	Univariable	Multivariable
*Interpersonal*						
3–4 year-olds enrolled at setting (% of total)	− 0.0 (− 0.1, 0.0)	–	− 0.0 (− 0.0, 0.0)	–	0.0 (− 0.1, 0.1)	–
Class composition (3–4 yrs) (ref: 2–4 yrs)	0.4 (− 1.6, 2.4)	–	1.5 (− 0.1, 3.2)	–	− 1.8 (− 4.6, 1.0)	–
Government funded places	− 0.0 (− 0.1, 0.0)	–	− 0.0 (− 0.0, 0.0)	–	− 0.0 (− 0.0, 0.0)	–
Children per staff member^a^	− 4.3 (− 14.7, 6.2)	–	3.7 (− 4.4, 11.8)	–	− 0.7 (− 12.6, 11.3)	–
Staff mean age in years	− 0.1 (− 0.2, 0.1)	–	0.0 (− 0.1, 0.1)	–	0.1 (− 0.1, 0.2)	–
Staff mean years at setting	0.2 (− 0.1, 0.6)	–	0.2 (− 0.1, 0.5)	–	− 0.4 (− 0.9, 0.1)	–
Staff mean years in childcare	− 0.1 (0.3, 0.1)	–	0.1 (− 0.1, 0.2)	–	0.1 (− 0.2, 0.3)	–
Staff behaviour§						

*Environmental*						
Active opportunities§	1.9 (0.9, 2.9)^⁎⁎⁎^	1.9 (0.9, 2.9) ^⁎⁎⁎^	− 0.8 (− 1.7, 0.1)	–	− 1.1 (− 2.6, 0.4)	–
Sedentary opportunities	0.1 (− 1.4, 1.6)	–	− 0.4 (− 1.6, 0.9)	–	0.2 (− 1.9, 2.2)	–
Fixed equipment§	− 0.1 (− 0.5, 0.2)	–	− 0.2 (− 0.5, 0.1)	–	0.4 (− 0.1, 0.9)	–
Portable equipment§	− 0.2 (− 0.5, 0.1)	–	0.2 (− 0.1, 0.4)	–	0.0 (− 0.4, 0.5)	–
Sedentary environment§	0.2 (− 0.1, 0.4)	–	− 0.1 (− 0.3, 0.2)	–	− 0.1 (− 0.5, 0.2)	–
Time allowed outside¥ (%)	− 0.0 (− 0.1, 0.0)	–	− 0.0 (− 0.1, 0.0)	–	− 0.0 (− 0.1, 0.1)	–
Time children seated¥ (%)	0.1 (− 0.0, 0.2)	–	− 0.0 (− 0.1, 0.1)	–	− 0.1 (− 0.2, 0.1)	–
Reported time spent in GMP (ref: 0–60)						
61–120	1.2 (− 2.0, 4.4)	–	− 0.4 (− 3.1, 2.3)	–	− 0.7 (− 5.3, 3.8)	–
121–180	3.1 (0.4, 7.0)^⁎^	ns	− 1.4 (− 4.2, 1.4)	–	− 2.4 (− 7.0, 2.2)	–
181 +	3.4 (0.4, 6.4)^⁎^	ns	− 1.1 (− 3.6, 1.4)	–	− 2.3 (− 6.5, 1.9)	–

Exposure	Outcome β (95% CI)					

SED		LPA		MVPA	

Univariable	Multivariable	Univariable	Multivariable	Univariable	Multivariable

Policy						
Physical activity training and education§	0.8 (− 0.5, 2.1)	–	0.7 (− 0.4, 1.8)	–	− 1.3 (− 3.1, 0.5)	–
Physical activity policies§	− 1.3 (− 2.8, 0.2)	–	1.1 (− 0.2, 2.3)	–	0.3 (− 1.8, 2.4)	–
Play outside in light rain: (ref: clothes)						
Always	− 0.7 (− 3.4, 1.9)	–	0.2 (− 2.1, 2.4)	–	0.7 (− 3.0, 4.4)	–
Play outside in heavy rain: (ref: with clothes)						
Always	− 0.1 (− 3.1, 2.9)	–	− 0.1 (− 2.5, 2.4)	–	0.1 (− 4.1, 4.2)	–
Play outside in snow: (ref: with clothes)						
Always	2.1 (0.1, 4.0)^⁎^	ns	− 0.9 (− 2.6, 0.7)	–	− 1.1 (− 3.8, 1.6)	–
Play outside in sun: (ref: with clothes)						
Always	− 1.2 (− 5.3, 2.9)	–	2.9 (− 3.9, 9.0)	–	− 1.9 (− 16.2, 12.3)	–
Ofsted score¥ (ref: satisfactory)						
Good/outstanding	1.4 (− 1.3, 3.2)	–	− 1.4 (− 3.7, 0.9)	–	− 0.1 (− 4.0, 3.8)	–
Between child variance (mean (std error))	4.50 (0.51)	3.82 (0.46)	7.59 (0.62)			
Within child variance (mean (std error))	8.44 (0.26)	7.72 (0.23)	10.78 (0.33)			

β: Minutes of activity per hour in care; ‘–’: not entered into adjusted analyses; GMP: Gross Motor Play; all analyses adjusted for sex, daily hours in care and mode of travel to preschool: *p < 0.05; **p < 0.01; ***p < 0.001

a Calculated as a ratio: number of children in room/number of staff in room.

§ Denotes EPAO subscale score used.

¥ From setting observation; all other variables taken from the setting questionnaire; ns: not significant in adjusted analyses.
